# The genome sequence of the square-spot rustic, *Xestia xanthographa *(Schiffermuller, 1775)

**DOI:** 10.12688/wellcomeopenres.17538.1

**Published:** 2022-02-02

**Authors:** Douglas Boyes, Peter W.H. Holland, University of Oxford and Wytham Woods Genome Acquisition Lab, Darwin Tree of Life Barcoding collective, Wellcome Sanger Institute Tree of Life programme, Wellcome Sanger Institute Scientific Operations: DNA Pipelines collective, Tree of Life Core Informatics collective, Darwin Tree of Life Consortium

**Affiliations:** 1 UK Centre for Ecology and Hydrology, Wallingford, Oxfordshire, UK; 2 Department of Zoology, University of Oxford, Oxfors, UK

**Keywords:** Xestia xanthographa, square-spot rustic, genome sequence, chromosomal, Lepidoptera

## Abstract

We present a genome assembly from an individual male *Xestia xanthographa *(the square-spot rustic; Arthropoda; Insecta; Lepidoptera; Noctuidae). The genome sequence is 934 megabases in span. The majority of the assembly (99.94%) is scaffolded into 31 chromosomal pseudomolecules, with the Z sex chromosome assembled.

## Species taxonomy

Eukaryota; Metazoa; Ecdysozoa; Arthropoda; Hexapoda; Insecta; Pterygota; Neoptera; Endopterygota; Lepidoptera; Glossata; Ditrysia; Noctuoidea; Noctuidae; Noctuinae; Noctuini; Xestiai; *Xestia xanthographa* (Schiffermuller, 1775) (NCBI:txid988049).

## Background

*Xestia xanthographa* (square-spot rustic) is a widespread noctuid moth found across much of the Palearctic, Europe, North Africa and North America; its larvae are nocturnal feeders on various grasses. In the UK, adults are abundant in late summer from August to September and the species overwinters as a larva. *Xestia xanthographa* was a key species in a recent study revealing the detrimental effects of street-lighting on caterpillar abundance in the UK ( Boyes *et al.*, 2021). The species has also been recorded as a common prey species for autumn-flying bats ( Razgour *et al.*, 2011), and, as an adaptation to facilitate bat avoidance, the auditory sensitivity of *X. xanthographa* is broadly tuned with an optimal frequency of 30 kHz ( Norman & Jones, 2008).

The genome of *X. xanthographa*, was sequenced as part of the Darwin Tree of Life Project, a collaborative effort to sequence all of the named eukaryotic species in the Atlantic Archipelago of Britain and Ireland. Here we present a chromosomally complete genome sequence for *X. xanthographa*, based on one male specimen from Wytham Woods, Oxfordshire, UK.

## Genome sequence report

**Figure 1.**
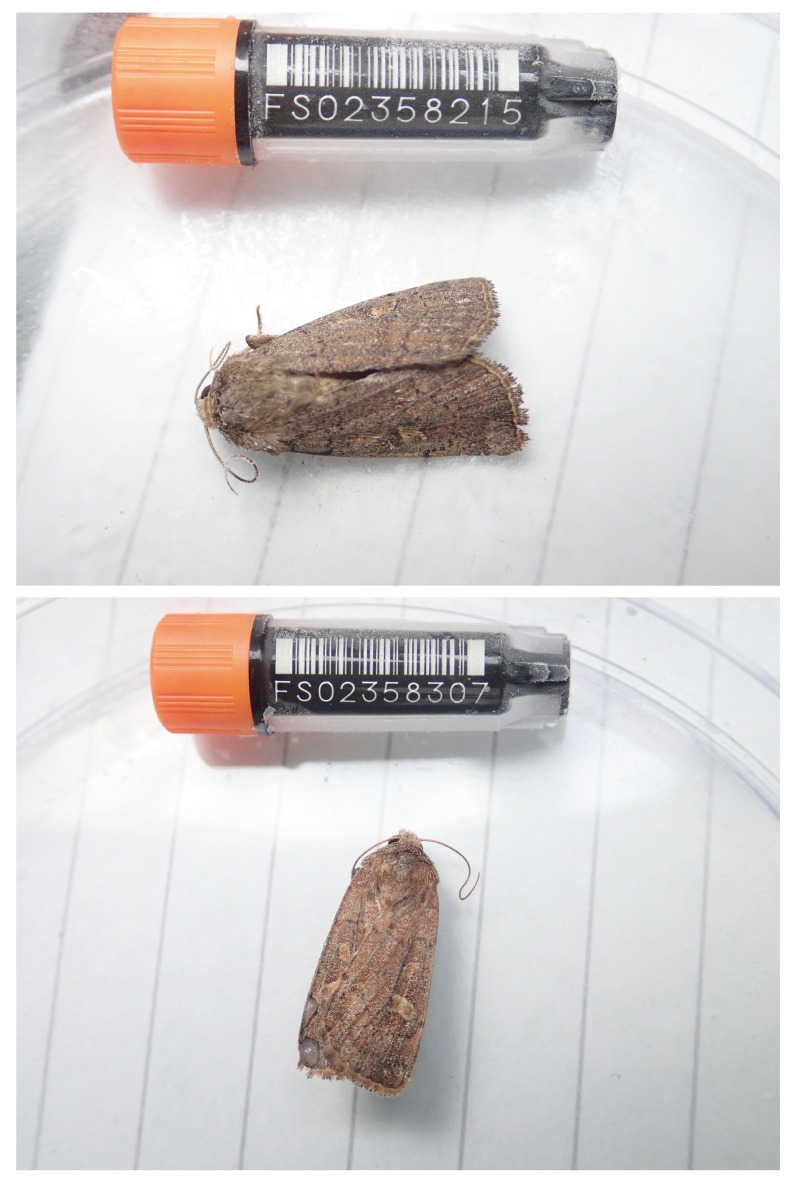
Image of the *Xestia xanthographa* specimens taken prior to preservation and processing. Above, ilXesXant, used for genome and Hi-C sequencing; below, ilXesXant2, used for RNA-Seq.

The genome was sequenced from a single male *X. xanthographa* collected from Wytham Woods, Oxfordshire, UK (latitude 51.772, longitude -1.337) ( Figure 1). A total of 27-fold coverage in Pacific Biosciences single-molecule long reads (N50 12 kb) and 40-fold coverage in 10X Genomics read clouds were generated. Primary assembly contigs were scaffolded with chromosome conformation Hi-C data. Manual assembly curation corrected 86 missing/misjoins and removed 17 haplotypic duplications, reducing the assembly size by 1.41% and scaffold number by 44.86%, and increasing the scaffold N50 by 1.61%.

**Figure 2.**
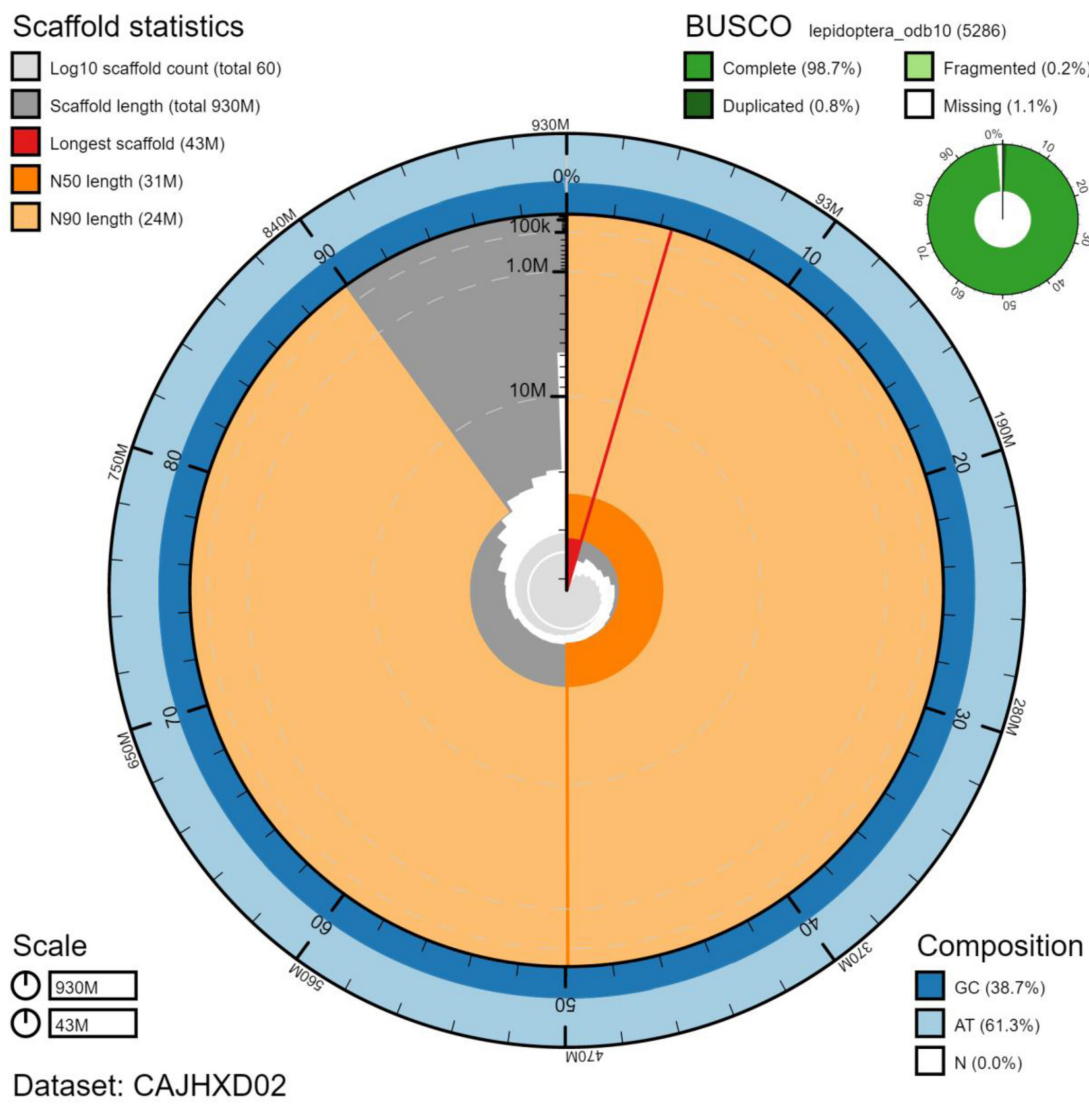
Genome assembly of *Xestia xanthographa*, ilXesXant1.2: metrics. The BlobToolKit Snailplot shows N50 metrics and BUSCO gene completeness. The main plot is divided into 1,000 size-ordered bins around the circumference with each bin representing 0.1% of the 933,882,218 bp assembly. The distribution of scaffold lengths is shown in dark grey with the plot radius scaled to the longest scaffold present in the assembly (42,568,189 bp, shown in red). Orange and pale-orange arcs show the N50 and N90 scaffold lengths (31,247,186 and 23,512,362 bp), respectively. The pale grey spiral shows the cumulative scaffold count on a log scale with white scale lines showing successive orders of magnitude. The blue and pale-blue area around the outside of the plot shows the distribution of GC, AT and N percentages in the same bins as the inner plot. A summary of complete, fragmented, duplicated and missing BUSCO genes in the lepidoptera_odb10 set is shown in the top right. An interactive version of this figure is available at https://blobtoolkit.genomehubs.org/view/ilXesXant1.2/dataset/CAJHXD02/snail.

The final assembly has a total length of 934 Mb in 59 sequence scaffolds with a scaffold N50 of 31 Mb ( Table 1). Of the assembly sequence, 99.94% was assigned to 31 chromosomal-level scaffolds, representing 30 autosomes (numbered by sequence length), and the Z sex chromosome ( Figure 2– Figure 5; Table 2). The assembly has a BUSCO ( Simão *et al.*, 2015) completeness of 98.7% using the lepidoptera_odb10 reference set. While not fully phased, the assembly deposited is of one haplotype. Contigs corresponding to the second haplotype have also been deposited.

**Table 1.** Genome data for *Xestia xanthographa*, ilXesXant1.2.

*Project accession data*	
Assembly identifier	ilXesXant1.2
Species	*Xestia xanthographa*
Specimen	ilXesXant1
NCBI taxonomy ID	NCBI:txid988049
BioProject	PRJEB42066
BioSample ID	SAMEA7520195
Isolate information	Male, head/abdomen/thorax
*Raw data accessions*	
PacificBiosciences SEQUEL II	ERR6635594
10X Genomics Illumina	ERR6002560-ERR6002563
Hi-C Illumina	ERR6002564
Illumina polyA RNA-seq	ERR6002565, ERR6787402
*Genome assembly*	
Assembly accession	GCA_905147715.2
*Accession of alternate haplotype*	GCA_905147755.2
Span (Mb)	934
Number of contigs	301
Contig N50 length (Mb)	9.1
Number of scaffolds	59
Scaffold N50 length (Mb)	31.2
Longest scaffold (Mb)	35.5
BUSCO * genome score	C:98.7%[S:97.9%,D:0.8%], F:0.2%,M:1.1%,n:5286

*BUSCO scores based on the lepidoptera_odb10 BUSCO set using v5.1.2. C= complete [S= single copy, D=duplicated], F=fragmented, M=missing, n=number of orthologues in comparison. A full set of BUSCO scores is available at https://blobtoolkit.genomehubs.org/view/ilXesXant1.2/dataset/CAJHXD02/busco.

**Figure 3.**
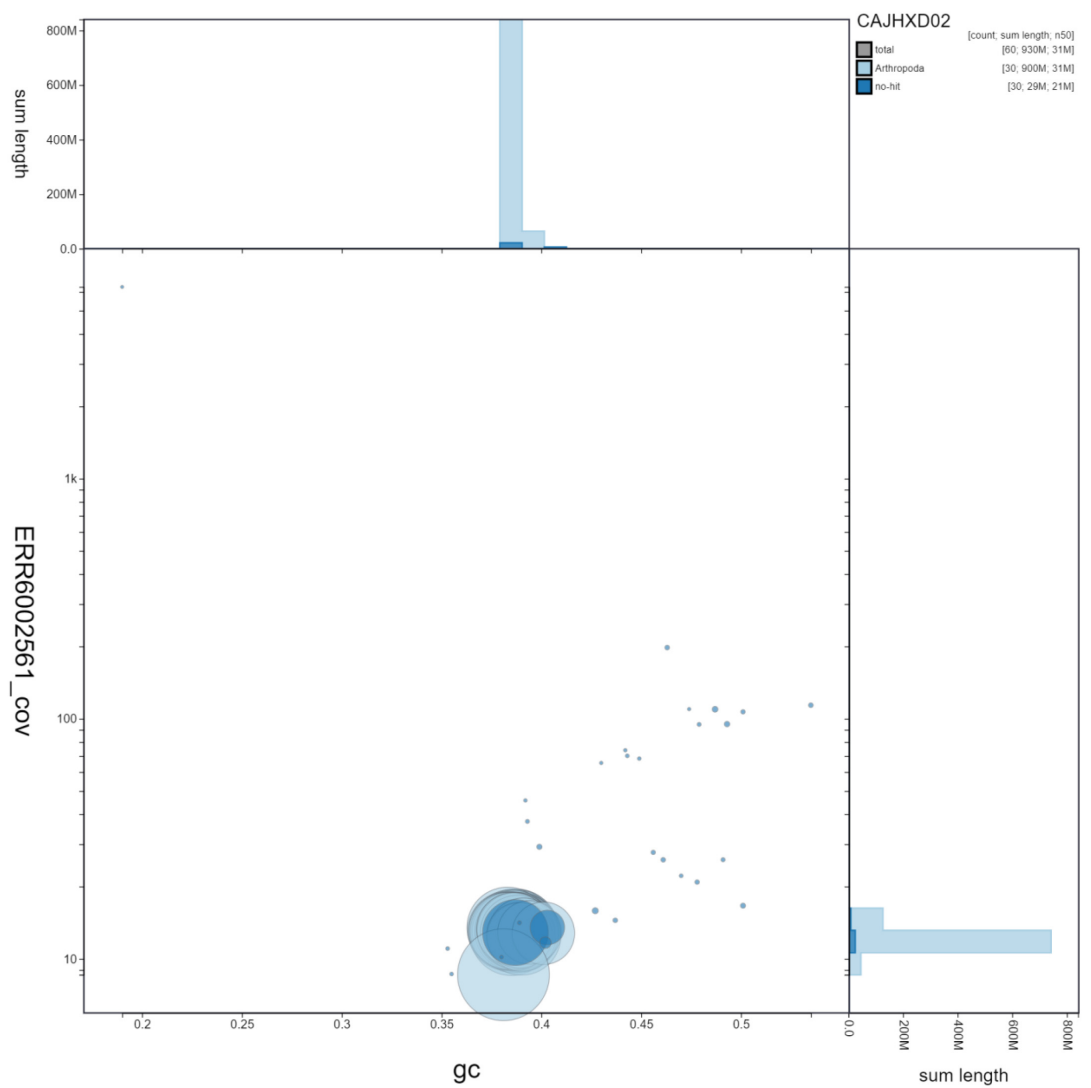
Genome assembly of *Xestia xanthographa*, ilXesXant1.2: GC coverage. BlobToolKit GC-coverage plot. Scaffolds are coloured by phylum. Circles are sized in proportion to scaffold length. Histograms show the distribution of scaffold length sum along each axis. An interactive version of this figure is available at https://blobtoolkit.genomehubs.org/view/ilXesXant1.2/dataset/CAJHXD02/blob.

**Figure 4.**
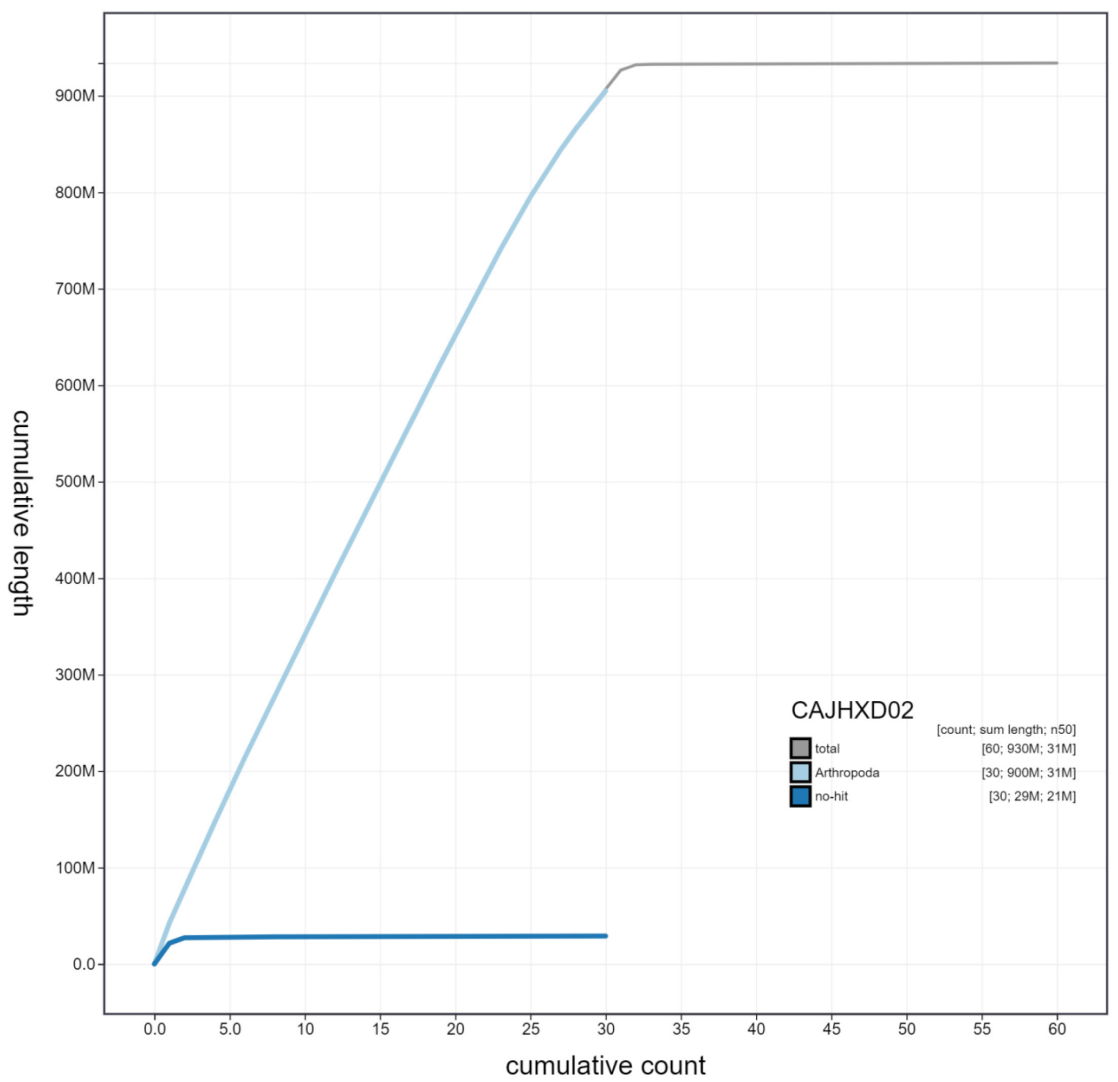
Genome assembly of *Xestia xanthographa*, ilXesXant1.2: cumulative sequence. BlobToolKit cumulative sequence plot. The grey line shows cumulative length for all scaffolds. Coloured lines show cumulative lengths of scaffolds assigned to each phylum using the buscogenes taxrule. An interactive version of this figure is available at https://blobtoolkit.genomehubs.org/view/ilXesXant1.2/dataset/CAJHXD02/cumulative.

**Figure 5.**
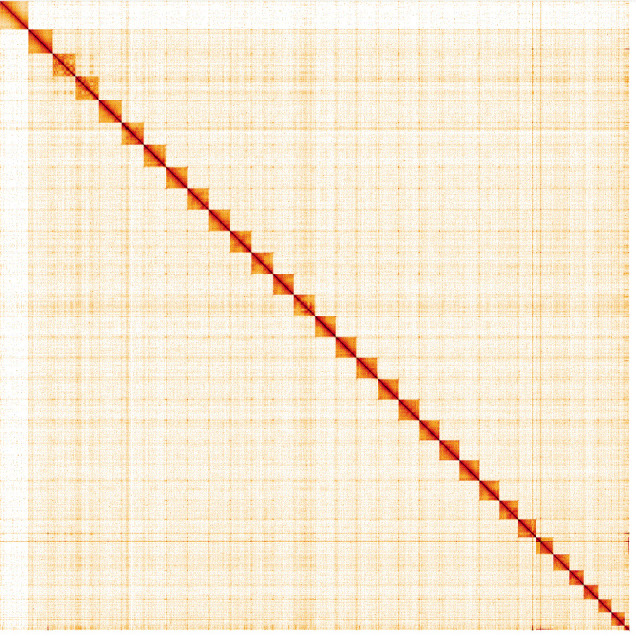
Genome assembly of *Xestia xanthographa*, ilXesXant1.2: Hi-C contact map. Hi-C contact map of the ilXesXant1.2 assembly, visualised in HiGlass. Chromosomes are given in size order from left to right and top to bottom.

**Table 2.** Chromosomal pseudomolecules in the genome assembly of *Xestia xanthographa*, ilXesXant1.2.

INSDC accession	Chromosome	Size (Mb)	GC%
LR990642.1	1	35.50	38.7
LR990643.1	2	34.37	38.5
LR990644.1	3	34.35	38.8
LR990645.1	4	33.93	38.7
LR990646.1	5	32.94	38.3
LR990647.1	6	32.23	38.5
LR990648.1	7	32.15	38.6
LR990649.1	8	32.03	38.9
LR990650.1	9	31.69	38.7
LR990651.1	10	31.50	38.9
LR990652.1	11	31.49	38.8
LR990653.1	12	31.35	38.3
LR990654.1	13	31.25	39
LR990655.1	14	31.05	38.6
LR990656.1	15	30.95	38.5
LR990657.1	16	30.88	38.5
LR990658.1	17	30.80	38.7
LR990659.1	18	30.66	38.4
LR990660.1	19	30.04	38.7
LR990661.1	20	29.75	38.6
LR990662.1	21	29.67	38.3
LR990663.1	22	29.39	38.7
LR990664.1	23	27.72	38.8
LR990665.1	24	27.14	38.6
LR990666.1	25	24.91	39.1
LR990667.1	26	23.51	39
LR990668.1	27	21.65	38.9
LR990669.1	28	21.50	38.7
LR990670.1	29	20.05	39.4
LR990671.1	30	19.43	40.1
LR990641.1	Z	42.57	38.1
LR990672.1	MT	0.02	19.2
-	-	7.42	41.1

## Methods

### Sample acquisition and nucleic acid extraction

A male *X. xanthographa* (ilXesXant1) and a second specimen of unknown sex (ilXesXant2) were collected from Wytham Woods, Oxfordshire, UK (latitude 51.772, longitude -1.337) by Douglas Boyes, University of Oxford, using a light trap. The specimens were identified by the same individual and snap-frozen on dry ice.

DNA was extracted from whole organism tissue of ilXesXant1 at the Wellcome Sanger Institute (WSI) Scientific Operations core from the whole organism using the Qiagen MagAttract HMW DNA kit, according to the manufacturer’s instructions. RNA was extracted from thorax/abdomen tissue of ilXesXant2 in the Tree of Life Laboratory at the WSI using TRIzol (Invitrogen), according to the manufacturer’s instructions. RNA was then eluted in 50 μl RNAse-free water and its concentration assessed using a Nanodrop spectrophotometer and Qubit Fluorometer using the Qubit RNA Broad-Range (BR) Assay kit. Analysis of the integrity of the RNA was done using Agilent RNA 6000 Pico Kit and Eukaryotic Total RNA assay.

### Sequencing

Pacific Biosciences HiFi circular consensus and 10X Genomics Chromium read cloud sequencing libraries were constructed according to the manufacturers’ instructions. Poly(A) RNA-Seq libraries were constructed using the NEB Ultra II RNA Library Prep kit. Sequencing was performed by the Scientific Operations core at the Wellcome Sanger Institute on Pacific Biosciences SEQUEL II (HiFi), Illumina HiSeq X (10X) and Illumina HiSeq 4000 (RNA-Seq) instruments. Hi-C data were generated from abdomen tissue of ilXesXant1 using the Arima v1.0 kit and sequenced on HiSeq X.

### Genome assembly

Assembly was carried out with Hifiasm ( Cheng *et al.*, 2021). Haplotypic duplication was identified and removed with purge_dups ( Guan *et al.*, 2020). One round of polishing was performed by aligning 10X Genomics read data to the assembly with longranger align, calling variants with freebayes ( Garrison & Marth, 2012). The assembly was then scaffolded with Hi-C data ( Rao *et al.*, 2014) using SALSA2 ( Ghurye *et al.*, 2019). The assembly was checked for contamination and corrected using the gEVAL system ( Chow *et al.*, 2016) as described previously ( Howe *et al.*, 2021). Manual curation was performed using gEVAL, HiGlass ( Kerpedjiev *et al.*, 2018) and Pretext. The mitochondrial genome was assembled using MitoHiFi ( Uliano-Silva *et al.*, 2021), which performed annotation using MitoFinder ( Allio *et al.*, 2020). The genome was analysed and BUSCO scores generated within the BlobToolKit environment ( Challis *et al.*, 2020). Table 3 contains a list of all software tool versions used, where appropriate.

**Table 3.** Software tools used.

Software tool	Version	Source
HiCanu	1.0	Cheng *et al.*, 2021
purge_dups	1.2.3	Guan *et al.*, 2020
SALSA2	2.2	Ghurye *et al.*, 2019
longranger align	2.2.2	https://support.10xgenomics.com/ genome-exome/software/pipelines/latest/ advanced/other-pipelines
freebayes	1.3.1-17-gaa2ace8	Garrison & Marth, 2012
MitoHiFi	1.0	Uliano-Silva *et al.*, 2021
gEVAL	N/A	Chow *et al.*, 2016
PretextView	0.1.x	https://github.com/wtsi-hpag/PretextView
HiGlass	1.11.6	Kerpedjiev *et al.*, 2018
BlobToolKit	2.6.4	Challis *et al.*, 2020

## Data availability

European Nucleotide Archive: Xestia xanthographa (square-spot rustic). Accession number PRJEB42066; https://identifiers.org/ena.embl/PRJEB42066.

The genome sequence is released openly for reuse. The *X. xanthographa* genome sequencing initiative is part of the Darwin Tree of Life (DToL) project. All raw sequence data and the assembly have been deposited in INSDC databases. The genome will be annotated and presented through the Ensembl pipeline at the European Bioinformatics Institute. Raw data and assembly accession identifiers are reported in Table 1.

## Author information

Members of the University of Oxford and Wytham Woods Genome Acquisition Lab are listed here: https://doi.org/10.5281/zenodo.5746938.

Members of the Darwin Tree of Life Barcoding collective are listed here: https://doi.org/10.5281/zenodo.5744972.

Members of the Wellcome Sanger Institute Tree of Life programme are listed here: https://doi.org/10.5281/zenodo.5744840.

Members of Wellcome Sanger Institute Scientific Operations: DNA Pipelines collective are listed here: https://doi.org/10.5281/zenodo.5746904.

Members of the Tree of Life Core Informatics collective are listed here: https://doi.org/10.5281/zenodo.5743293.

Members of the Darwin Tree of Life Consortium are listed here: https://doi.org/10.5281/zenodo.5638618.
