# Supplementary material for: The genome sequence of the square-spot rustic, Xestia xanthographa (Schiffermuller, 1775)
Source: Wellcome Open Res. 2022 Feb 2;7:37. [Version 1] doi: 10.12688/wellcomeopenres.17538.1 (PMC9664021; doi:10.12688/wellcomeopenres.17538.1)
Supplement: Peer review history

# Peer review: Reviewer response for version 1

**DOI:** 10.21956/wellcomeopenres.19393.r53110

**Published:** 2022-11-14

**Authors:** Steven M. Van Belleghem

**Affiliations:** 1 Department of Biology, Rio Piedras Campus, University of Puerto Rico, San Juan, Puerto Rico

Boyes and Holland report on the genome assembly of the square-spot rustic, Xestia xanthographa. They used Pacbio HiFi and 10X Genomics reads and Hi-C for scaffolding the contigs into chromosomes, resulting in a 934 Mb genome assembled into 31 chromosomes.

 The sequencing methods used are high-end, the assembly and QC methods are appropriate and the assembly seems of high quality.

Are sufficient details of methods and materials provided to allow replication by others?

Yes

Is the rationale for creating the dataset(s) clearly described?

Yes

Are the datasets clearly presented in a useable and accessible format?

Yes

Are the protocols appropriate and is the work technically sound?

Yes

Reviewer Expertise:

Adaptation, speciation, genomics

I confirm that I have read this submission and believe that I have an appropriate level of expertise to confirm that it is of an acceptable scientific standard.

# Peer review: Reviewer response for version 1

**DOI:** 10.21956/wellcomeopenres.19393.r48473

**Published:** 2022-02-09

**Authors:** Xin Liu

**Affiliations:** 1 State Key Laboratory of Agricultural Genomics, BGI (Beijing Genomics Institute)-Shenzhen, Shenzhen, China

The data note by Douglas *et al. *described the dataset of the assembled square-spot rustic genome. The manuscript clearly presented the methods used for sampling, sample preparation, sequencing, and data analysis, with the related statistics presented. The genome assembly is of good quality, with long scaffold N50 and anchored to chromosomes, making it valuable for later genome and other studies. I think the current manuscript is good enough to be published as a data note. Meanwhile, I have the following suggestions for the authors to consider:

1. In the ‘Genome sequence report’ section on Page 5, the statement of BUSCO results might need to be modified. Interpretation of the BUSCO result should be like “98.7% of the BUSCOs in the lepidoptera_odb10 dataset were found to be complete”, as you can also observe the other 1% to be either in multiple copies (duplicated), or fragmented. This reflected the completeness of the genome assembly.
2. Also, in the part mentioned above, I would suggest providing some statistics (at least the length maybe) of the assembled second haplotype, which would be informative for understanding the heterozygosity in the genome.
3. I found all the data notes in the same gateway directly used figures from the BlobToolkit Viewer. I would suggest revising the figures in the data note thus they would be a better fit. For example, the axis labels might be better to be revised in cases and the font sizes and the dataset label can also be removed. I don’t know what the ‘Scale’ means in Figure 2, and I think Figure 3 and Figure 4 are quite difficult to understand, at least for me.

Last, I would like to express my condolence on Douglas Boyes’ death. After noting this from the manuscript and searched online, I would like to thank his contributions in providing this valuable dataset.

Are sufficient details of methods and materials provided to allow replication by others?

Yes

Is the rationale for creating the dataset(s) clearly described?

Yes

Are the datasets clearly presented in a useable and accessible format?

Yes

Are the protocols appropriate and is the work technically sound?

Yes

Reviewer Expertise:

Genomics

I confirm that I have read this submission and believe that I have an appropriate level of expertise to confirm that it is of an acceptable scientific standard.
